# Letter from Darmstadt

**Published:** 1884-12

**Authors:** Sarah Hackett Stevenson

**Affiliations:** Darmstadt


### FOREIGN CORRESPONDENCE.

LETTER FROM DARMSTADT.

DARMSTADT, Oct. 6, 1884.

One can not go far or stay long in Germany without meeting some great medical light. There is scarcely a town of any size, even though it contain no university, that can not boast of some great teacher.

Having a day to spend here with my former pupil and friend, Dr. Fanny Dickinson, I was introduced to the celebrated Dr. Weber, the friend and peer of Von Graefe.

Here, in this quiet, sleepy town, lives this genius of our art, having no desire to go to the world, for the world comes to him. I was truly glad to know that in the midst of his great practice and his numerous researches he found the time and had the graciousness to patiently instruct a young American girl in his own incomparable methods. Doing this without seeming arrogance or condescension would, of itself, constitute a sign of greatness.

Although my visit was purely social, yet I did not fail to receive a great many scientific suggestions. As Darmstadt is likewise the home of Merck and his great laboratory, or "Fabrik," very many of the new alkaloids are sent to Dr. Weber for experiment. I found the Doctor in the midst of his experiments with "cocain," the alkaloid of "cocoa erythroxylon." The Doctor has found this to be more than a substitute for atropine, in that, while it dilates the pupil equally well, it does not disturb the accommodation, and it also has an anæsthetic effect, so that the most serious operations about the eye may possibly be performed without the use of chloroform or ether. Dr. Weber informed me that while his experiments with the drug are by no means complete, he hopes to find in it an anæsthetic for general as well as ophthalmic surgery. He had injected a solution into his own arm, and found sensation completely obliterated below the point. He was as enthusiastic over all this as an English boy would be over a new bicycle! And while he talked I observed that the pupil of his right eye was much larger than that of the left. He explained that he had used cocain in both eyes, and then eserin in the left eye; hence the disparagement. I had no idea that my call would prove so much of a clinic!

Accidentally, the conversation turned upon apomorphin, and the Doctor informed me that he had found it a most excellent remedy in chronic asthma, and related a case of some thirty years' standing that had been permanently cured by the administration of one-twelfth grain three times daily, gradually increasing the dose to one-third grain. The result was obtained in about three weeks. The Doctor's brother, who is an obstetrician, had found the remedy very useful in rigid os—not interfering in the least with the normal contractions. My attention was likewise called to a new stethoscope, invented by Bazille, upon the principle of a cupping-glass. It consisted of one hard-rubber cup within another, so arranged that the air could be exhausted from the space between the two cups, thereby deadening all external sounds. The fault in the instrument is, that in cases of much emaciation it would be impossible to apply the cup; and yet these are the cases in which fine shades in diagnosis are seldom needed, for usually, when there is great emaciation, the lesions are gross enough to be recognized without an instrument. It will certainly prove a valuable instrument in the incipient stages of lung diseases, and of still greater value in diseases of the heart.

But medicine is not the Doctor's only forte. He is well read in our best English authors, both literary and philosphic. He called my attention to a fine first-proof steel engraving of John Stuart Mill, presented to him by the Queen of England, and then showed me what he called his bible, which proved to be Mill's Logic. "This," he said, "I read the last thing at night and the first thing in the morning."

Through this genial genius, Dr. Weber, I was introduced to another as great a genius, in his way, and equally genial, Dr. Merck, of the Darmstadt alkaloid fame. He took us all through his great "fabrik," and explained most patiently and lucidly the various processes of preparing both the organic and inorganic substances. He called my attention to a new drug he was preparing from Helenium. This plant, as you remember, was named for Helen, wife of Menelaus, but which in our common English is known by the rare and euphonious name of "sneeze-weed." Alas for the classic Helen! Dr. Merck claims that this is an unparalleled remedy for the night-sweats of phthisis, but he could not at the time remember what physician had made the discovery.

And thus, one can not even visit in Germany without learning by the way; indeed, learning is in the very air, and it becomes a part of the respiratory process.

Begging your pardon for so much gossip, although pseudo-scientific, I have the honor to be,

Yours truly,

SARAH HACKETT STEVENSON.

